# Diagnostic Performance of Nanopore-targeted Sequencing for Sputum Smear-negative Pulmonary Tuberculosis: A Retrospective Study

**DOI:** 10.1093/ofid/ofag323

**Published:** 2026-05-26

**Authors:** Wei Wei, Xubin Zheng, Lan Yao, Qin Sun, Wei Sha

**Affiliations:** Clinic and Research Centre of Tuberculosis, Shanghai Key Laboratory of Tuberculosis, Shanghai Pulmonary Hospital, School of Medicine, Tongji University, Shanghai, China; Clinic and Research Centre of Tuberculosis, Shanghai Key Laboratory of Tuberculosis, Shanghai Pulmonary Hospital, School of Medicine, Tongji University, Shanghai, China; Division of Infectious Diseases, Department of Medicine, Karolinska Institute, Stockholm, Sweden; Department of Infectious Diseases, Karolinska University Hospital, Stockholm, Sweden; Clinic and Research Centre of Tuberculosis, Shanghai Key Laboratory of Tuberculosis, Shanghai Pulmonary Hospital, School of Medicine, Tongji University, Shanghai, China; Clinic and Research Centre of Tuberculosis, Shanghai Key Laboratory of Tuberculosis, Shanghai Pulmonary Hospital, School of Medicine, Tongji University, Shanghai, China; Clinic and Research Centre of Tuberculosis, Shanghai Key Laboratory of Tuberculosis, Shanghai Pulmonary Hospital, School of Medicine, Tongji University, Shanghai, China

**Keywords:** diagnosis, *M. tuberculosis*, nanopore-targeted sequencing, pulmonary disease, read-count cutoff

## Abstract

**Purpose:**

To evaluate the diagnostic performance of nanopore-targeted sequencing (NTS) for sputum smear-negative pulmonary tuberculosis (PTB).

**Methods:**

In this retrospective study, bronchoalveolar lavage fluid from patients with suspected PTB and negative sputum smears was tested with NTS, Xpert MTB/RIF, and MGIT 960 culture at Shanghai Pulmonary Hospital (August 2022–March 2024). Sensitivity and specificity were evaluated against both microbiological and composite reference standards. Receiver operating characteristic curves were generated and Youden's index was employed to determine the optimal NTS cutoff.

**Results:**

Among the 249 participants enrolled, 100 were microbiologically confirmed PTB, 33 were probable PTB, and 116 were tuberculosis-negative. Under microbiological reference standard, the sensitivity of NTS was 0.940 (95% confidence interval [CI], 0.869–0.975), higher than Xpert MTB/RIF (0.840 [95% CI, 0.750–0.903]; *P* = .041) and MGIT 960 culture (0.640 [95% CI, 0.537–0.732]; both *P* < .001). The specificity of NTS was 0.776 (95% CI 0.687–0.846), compared to 0.974 (95% CI 0.921–0.993) for Xpert MTB/RIF and 1.000 (95% CI 0.960–1.000) for MGIT 960 culture. Excluding patients with prior PTB history, NTS specificity increased to 0.856 (95% CI, 0.766–0.916). According to Youden's index, when 4 *Mycobacterium tuberculosis*–specific sequence reads was defined as the optimal NTS cutoff for positive NTS result, the specificity of NTS rose to 0.907 (95% CI, 0.827–0.954).

**Conclusions:**

NTS offers rapid, highly sensitive detection of PTB in patients with low bacillary burden. Applying an optimal read-count cutoff and accounting for prior PTB history can enhance clinical utility.

## Graphical Abstract

**Graphical Abstract:**
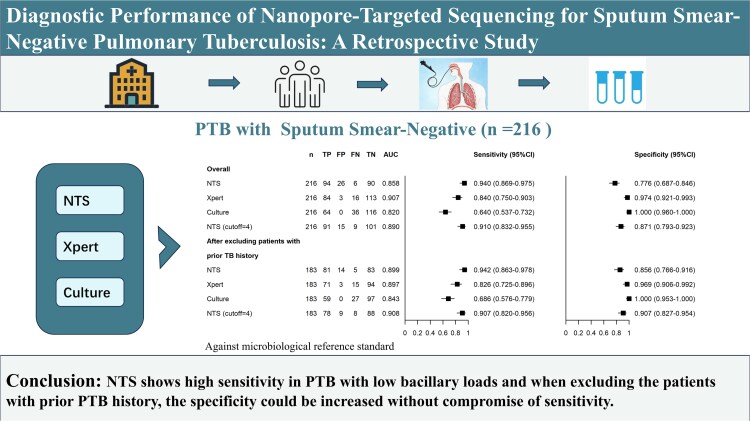

Pulmonary tuberculosis (PTB) remains a major global health threat despite the availability of improved diagnostic tools. Globally, in 2022, it was estimated that 10.6 million people suffered from tuberculosis, but only 7.5 million (70%) were diagnosed [1]. Xpert MTB/RIF, 1 of the rapid diagnostic tools for *Mycobacterium tuberculosis* (MTB) detection, plays a crucial role in improving detection rates, shortening diagnosis time, and identifying rifampicin resistance [2, 3]. In contrast to whole-genome sequencing, Xpert MTB/RIF is restricted to detecting *M. tuberculosis* and demonstrates relatively poor sensitivity (28%–46%) for PTB patients with sputum smear-negative and culture-positive results [4, 5].

Since 2004, metagenomic next-generation sequencing has emerged as an enabling technological platform for detecting microorganisms in clinical samples [6], showing excellent diagnostic performance in both tuberculosis and nontuberculous mycobacteria [7–9]. Targeted next-generation sequencing, combining multiplex polymerase chain reaction (PCR) with high-throughput sequencing, also plays a key role in rapid and accurate diagnosis of tuberculosis [10]. Nonetheless, implementation of NGS in routine clinical laboratories remains constrained by platform complexity and the need for specialized technical and bioinformatics expertise [11].

Nanopore-targeted sequencing (NTS) is a targeted third-generation sequencing technology that integrates ultra-multiplex PCR amplification with high-throughput nanopore sequencing. It can sequence and analyze nucleic acids in real time based on the electrical signals produced when different nucleotide bases pass through a nanopore [12, 13]. It features high-sensitivity, real-time sequencing results, portability, and long-read sequencing capabilities [14] and shows good comprehensive application value in clinical microbiological testing [15–18]. Previous studies have shown that NTS demonstrates higher sensitivity than Xpert MTB/RIF in detecting *M. tuberculosis* [19–21].

However, the sample size for previous studies was generally small and the evaluation relies primarily on composite reference standards. To date, few studies have specifically addressed the optimal cutoff threshold of NTS for defining a positive NTS result [22]. Meanwhile, few studies have paid attention to the factors affecting the specificity of NTS. Therefore, this study aimed to assess the diagnostic performance of NTS using bronchoalveolar lavage fluid (BALF) in patients with sputum smear-negative, to explore the factors affecting the NTS specificity and to determine the optimal read-count cutoff for NTS positivity.

## MATERIALS AND METHODS

### Study Design and Participants

A retrospective analysis was performed on patients with suspected active pulmonary tuberculosis and negative sputum smears at the Department of Tuberculosis, Tongji University Affiliated Shanghai Pulmonary Hospital (Shanghai, China), from August 2022 to March 2024. All participants underwent bronchoscopy, and BALF samples were subjected to NTS, Xpert MTB/RIF, and MGIT 960 culture.

Inclusion criteria were: (1) age between 16 and 80 years, regardless of gender; (2) HIV-negative status; (3) 3 consecutive sputum samples negative for acid-fast bacilli on smear; (4) availability of BALF samples for NTS, Xpert MTB/RIF, and MGIT 960 culture; and (5) chest computed tomography (CT) findings consistent with active pulmonary tuberculosis (such as patchy infiltrates, consolidation, multiple nodules, single or multiple cavities, bronchial dissemination, or tree-in-bud pattern).

Exclusion criteria were: (1) missing results for NTS, Xpert MTB/RIF, or MGIT 960 culture from BALF samples; and (2) ongoing antituberculosis treatment.

### Diagnostic Criteria of PTB

Diagnostic performance was assessed against both microbiological and composite reference standards based on the World Health Organization (WHO) Definitions and reporting framework for tuberculosis, 2013 revision (updated in December 2014 and January 2020) [23]. Patients were categorized into 3 groups: confirmed PTB, probable PTB, and TB-negative.

Microbiologically confirmed PTB required at least 1 positive result from MGIT 960 culture, Xpert MTB/RIF, or other validated molecular assay, but NTS results were not considered as part of the microbiological reference standard because this technique was under evaluation in this study.

Probable PTB was defined by fulfilment of all the following: (1) exclusion of other pulmonary diseases; (2) chest CT consistent with PTB; (3) purified protein derivative (PPD) induration of ≥10 mm or positive interferon-γ release assay; and (4) radiological improvement after 2 or more months of empirical anti-TB therapy.

TB-negative individuals were those who had no microbiological or clinical evidence supporting a diagnosis of PTB. The composite reference standard included microbiologically confirmed and probable PTB.

In this study, the diagnostic cutoff threshold for a positive NTS result was initially set as the detection of at least 1 *M. tuberculosis*–specific read, consistent with previous studies and the WHO Consolidated Guidelines on Tuberculosis, Module 3: Diagnosis (2021) and its accompanying Operational Handbook.

Nontuberculous mycobacterial pulmonary disease (NTM-PD) was diagnosed according to the guidelines of American Thoracic Society/European Respiratory Society/European Society of Clinical Microbiology and Infectious Diseases/Infectious Diseases Society of America (ATS/ERS/ESCMID/IDSA) (2020) and the Guidelines of Chinese Medical Association for the Diagnosis and Treatment of Non-tuberculous mycobacteria (2020), fungal pulmonary infection was defined according to the “Treatment of Invasive Pulmonary Aspergillosis and Preventive and Empirical Therapy for Invasive Candidiasis in Adult Pulmonary and Critical Care Patients: An Official American Thoracic Society Clinical Practice Guideline (2025).” Pneumonia was defined according to the “Diagnosis and Management of Community-acquired Pneumonia. An Official American Thoracic Society Clinical Practice Guideline (2025).”

### Data Collection

For each patient enrolled in this study, data were collected from medical records, including demographic data (eg, gender, age), underlying comorbidities, clinical symptoms, chest CT, and laboratory results (including NTS, Xpert MTB/RIF, and MGIT 960 culture).

### Specimen Processing and Laboratory Methods

#### Nanopore-targeted Sequencing (Hangzhou ShengTing)

A total of 10 mL of BALF was collected, stored, and transported at <4 °C. An equal volume of dithiothreitol solution, 10 µL of proteinase K, and 5 µL of lysozyme were added. The mixture was homogenized using 0.05-mm zirconia beads. Subsequently, 400 µL of the sample was added to the extraction reagent in accordance with the manufacturer's protocol (Hangzhou Shengting). PCR-amplified products were barcoded, purified using magnetic beads at a 0.6× ratio, and eluted with 15 µL of nuclease-enzyme-free water. The yield and quality of the eluted product (1 µL) was performed using a Qubit fluorometer. PCR products with equal quantities and different barcodes were pooled, then the library was prepared using the ligation sequencing kit (ShengTing) per the manufacturer's instructions. A total of 100 ng of the prepared library was loaded onto either a MinION or GridION platform (Oxford Nanopore Technologies). Automated data processing was performed with analysis software (Nano TNGS V1.0) and a secure online application (TBseq Web App) with integrated databases for interpretation of results.

We included 2 negative controls in each batch to monitor the contamination, 1 for DNA extraction and 1 for PCR amplification. NTS results were reported only if both negative controls passed quality control.

#### MGIT 960 Culture (Becton, Dickinson and Company, USA)

Once a positive MGIT 960 culture was obtained, the isolate was subjected to species identification by both growth on *p*-nitrobenzoic acid-containing media and MBP 64 antigen detection. Drug susceptibility testing was performed using the BACTEC MGIT 960 SIRE kit (BD, Franklin Lakes, NJ, USA) according to the manufacturer's instructions.

#### Xpert MTB/RIF (Cepheid GeneXpert System, Sunnyvale, CA, USA)

The Xpert MTB/RIF assay was according to the manufacturer's protocol. Briefly, 2 mL of sample processing solution was added to the centrifuged BALF specimen, mixed thoroughly, and incubated for 15 minutes at room temperature after vortexing. The mixture was transferred into an Xpert MTB/RIF cartridge, which was loaded into the GeneXpert instrument. Results for detection of *M. tuberculosis* and rifampicin resistance were automatically generated within 2 hours. The assay also provided a semiquantitative estimation of the *M. tuberculosis* load, categorized as “high,” “medium,” “low,” or “very low,” based on the cycle threshold value.

### Statistical Analysis

Statistical analyses were performed using SPSS 26.0 software (IBM Corp., Chicago, IL, USA). Continuous variables with normal distribution are expressed as mean ± standard deviation; otherwise as median (interquartile range). Categorical variables are presented as frequencies and percentages. Receiver operating characteristic curves were constructed and areas under the curve (AUC) were calculated. The optimal read-count cutoff for positive NTS result was determined using the maximum Youden's index. Sensitivities and specificities of NTS, MGIT 960, and Xpert MTB/RIF were compared using the paired McNemar chi-squared test, with statistical significance set at *P* < .05.

## RESULTS

### Patient Characteristics

**Figure 1.**
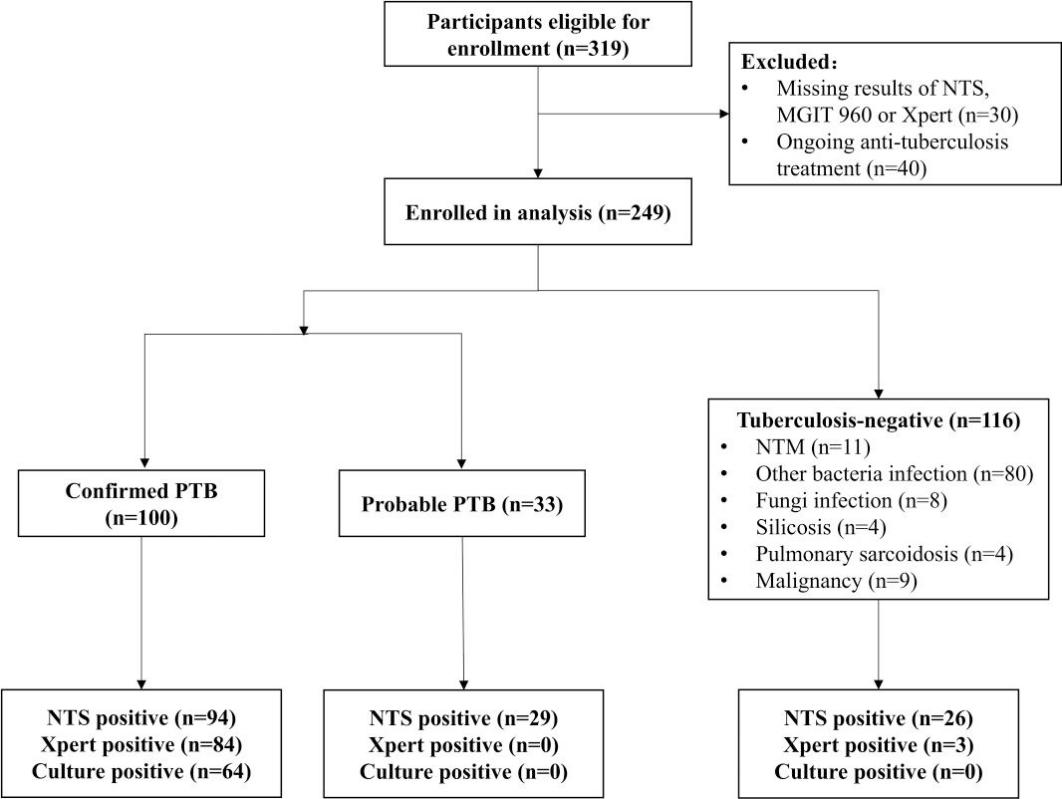
Study flowchart. Abbreviations: NTM, nontuberculous mycobacterial; NTS, nanopore-targeted sequencing.

Of 319 individuals screened, 249 were enrolled in this study. A total of 100 cases were classified as microbiologically confirmed PTB, 33 as probable PTB and 116 as TB-negative. Among the TB-negative controls, the final diagnoses included NTM-PD (n = 11), bacterial pneumonia/bronchiectasis (n = 80), malignancy (n = 9), pulmonary fungal infection (n = 8), silicosis (n = 4), and pulmonary sarcoidosis (n = 4) (Figure 1).

The median age of the participants was 52 years (±16.68), and 149 (59.6%) were male. A history of PTB was documented in 38 patients (15.2%) and diabetes mellitus in 21 (8.4%). Seventy-two patients (28.9%) were asymptomatic at admission. NTS yielded positive results in 149 of 249 patients (59.8%), compared to 87 (34.9%) for Xpert and 64 (25.7%) for MGIT 960 culture (Table 1).

**Table 1.** Demographic and Clinical Characteristics of the Included Patients

Categorize	All (n = 249)	Confirmed PTB (n = 100)	Probable PTB (n = 33)	Tuberculosis-negative (n = 116)
Age (year, mean ± SD)	52 ± 16.68	49.1 ± 18.00	47.7 ± 15.67	55.8 ± 14.90
Male (n, %)	149 (59.6)	68 (68.0)	16(48.5)	65 (56.0)
History of PTB (n, %)	38 (15.2)	14 (14.0)	5 (15.1)	19 (16.3)
QFT positive (n, %)	119 (48.0)	74 (74.0)	16 (48.4)	29 (25.0)
Comorbidities				
Diabetes (n, %)	21 (8.4)	7 (7.0)	6 (18.0)	8 (6.9)
Autoimmune disease (n, %)	4 (1.6)	2 (2.0)	1 (3.0)	1 (0.8)
Clinical characteristics				
Asymptomatic (n, %)	72 (28.9)	24 (24.0)	9 (27.2)	39 (33.6)
Cough (n, %)	118 (47.3)	47 (47.0)	19 (57.6)	52 (44.8)
Fever (n, %)	13 (5.2)	3 (3.0)	1 (3.0)	9 (7.7)
Hemoptysis (n, %)	16 (6.4)	5 (5.0)	2 (6.0)	9 (7.7)
Chest pain (n, %)	19 (7.6)	10 (10.0)	3 (9.0)	6 (5.2)
NTS positive (n)	149	94	29	26
Xpert positive (n)	87	84	0	3
High	1	1	0	0
Medium	16	16	0	0
Low	22	22	0	0
Very low	48	45	0	3
MGIT 960 positive (n)	64	64	0	0

Abbreviations: NTS, nanopore-targeted sequencing; PTB, pulmonary tuberculosis; QFT, QuantiFERON-TB Gold test; SD, standard deviation.

**Figure 2.**
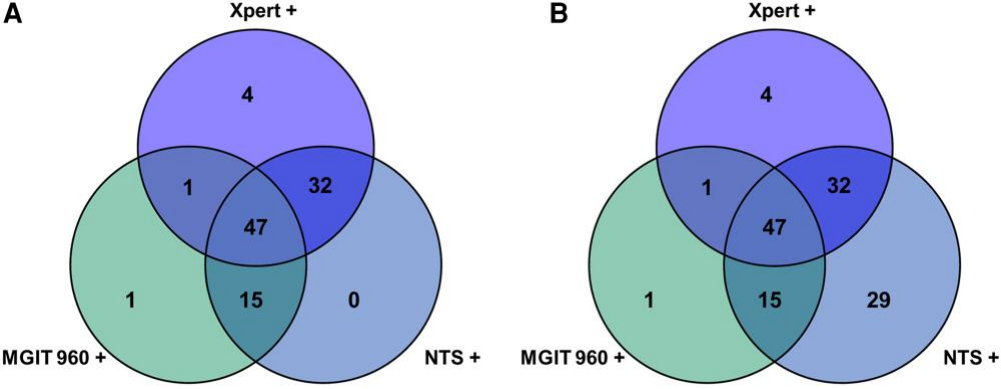
NTS, Xpert, and MGIT 960 results among patients with PTB against the microbiological reference standard (*A*) and the composite reference standard (*B*).

Consistency between NTS, Xpert, and MGIT 960 according to the microbiological reference standard, NTS was positive in 94 cases, Xpert MTB/RIF in 84 cases, and MGIT 960 culture in 64 cases (Figure 2*A*). Using the composite reference standard, NTS was positive in 123 cases, Xpert MTB/RIF in 84 cases, and MGIT 960 culture in 64 cases (Figure 2*B*). The distribution and overlap of positive results across the 3 diagnostic methods are illustrated in Figure 2.

### Comparisons of Diagnostic Performance of NTS, Xpert MTB/RIF, and MGIT 960 Culture

The median number of *M. tuberculosis*–specific reads detected by NTS among all 249 patients was 323 (range 1–33 498; interquartile range 11–3181).

**Figure 3.**
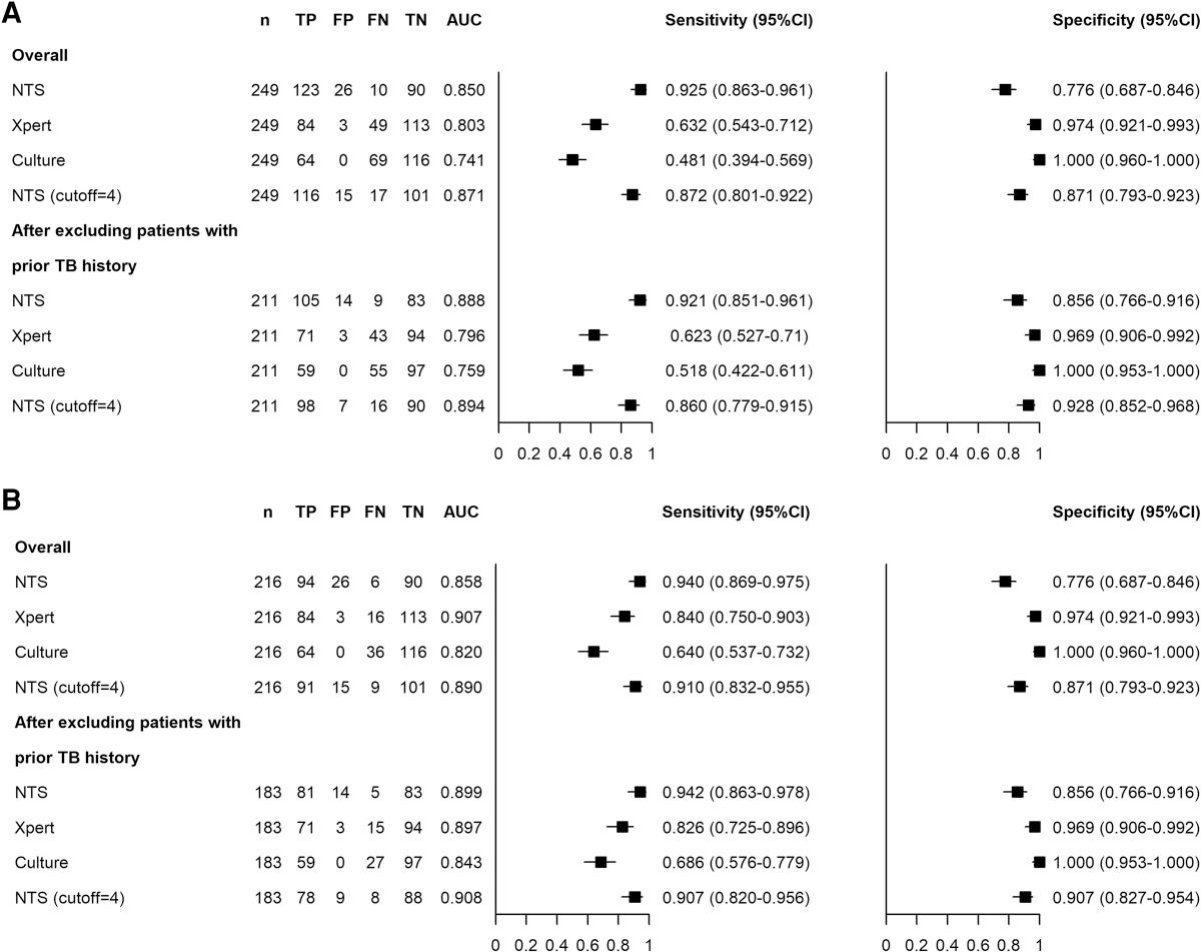
Sensitivity and specificity of NTS, Xpert, and culture diagnostic accuracy against the composite reference standard (*A*) and the microbiological reference standard (*B*) in patients with and without tuberculosis history. Abbreviations: FN, false negative; FP, false positive; TN, true negative; TP, true positive.

With the microbiological reference standard, NTS demonstrated a sensitivity of 0.940 (95% CI, 0.869–0.975), which was significantly higher than that of Xpert MTB/RIF (0.840 [95% CI 0.750–0.903]; *P* = .041) and MGIT 960 culture (0.640 [95% CI, 0.537–0.732]; *P* < .001). The specificity of NTS was 0.776 (95% CI, 0.687–0.846), which was lower than that of Xpert MTB/RIF (0.974 [95% CI, 0.921–0.993]; *P* < .001) and MGIT 960 culture (1.000 [95% CI, 0.960–1.000]) (Figure 3*B*). Using the composite reference standard, the sensitivity of NTS was 0.925 (95% CI, 0.863–0.961), compared to (0.632 [95% CI, 0.543–0.712]; *P* < .001) for Xpert MTB/RIF and (0.481 [95% CI, 0.394–0.569]; *P* < .001) for MGIT 960 culture (Figure 3*A*).

For the microbiological reference standard, the AUCs were 0.858 for NTS, 0.907 for Xpert MTB/RIF, and 0.820 for MGIT 960 culture. For the composite reference standard, the AUCs were 0.850 for NTS, 0.803 for Xpert MTB/RIF, and 0.741 for MGIT 960 culture.

### Potential Factors Affecting the Specificity of NTS

Among the 116 TB-negative patients, NTS detected *M. tuberculosis* in 26 cases and Xpert MTB/RIF detected *M. tuberculosis* in 3 cases (Figure 1). NTS-positive results mainly consisted of NTM-PD (n = 5), general bacterial infections (including bronchiectasis, chronic obstructive pulmonary disease, bacterial pneumonia, etc.) (n = 16), silicosis (n = 1), malignancy (n = 2), and fungal infection (n = 2). Notably, 12 of these 26 patients (46%) had a prior PTB history.

**Figure 4.**
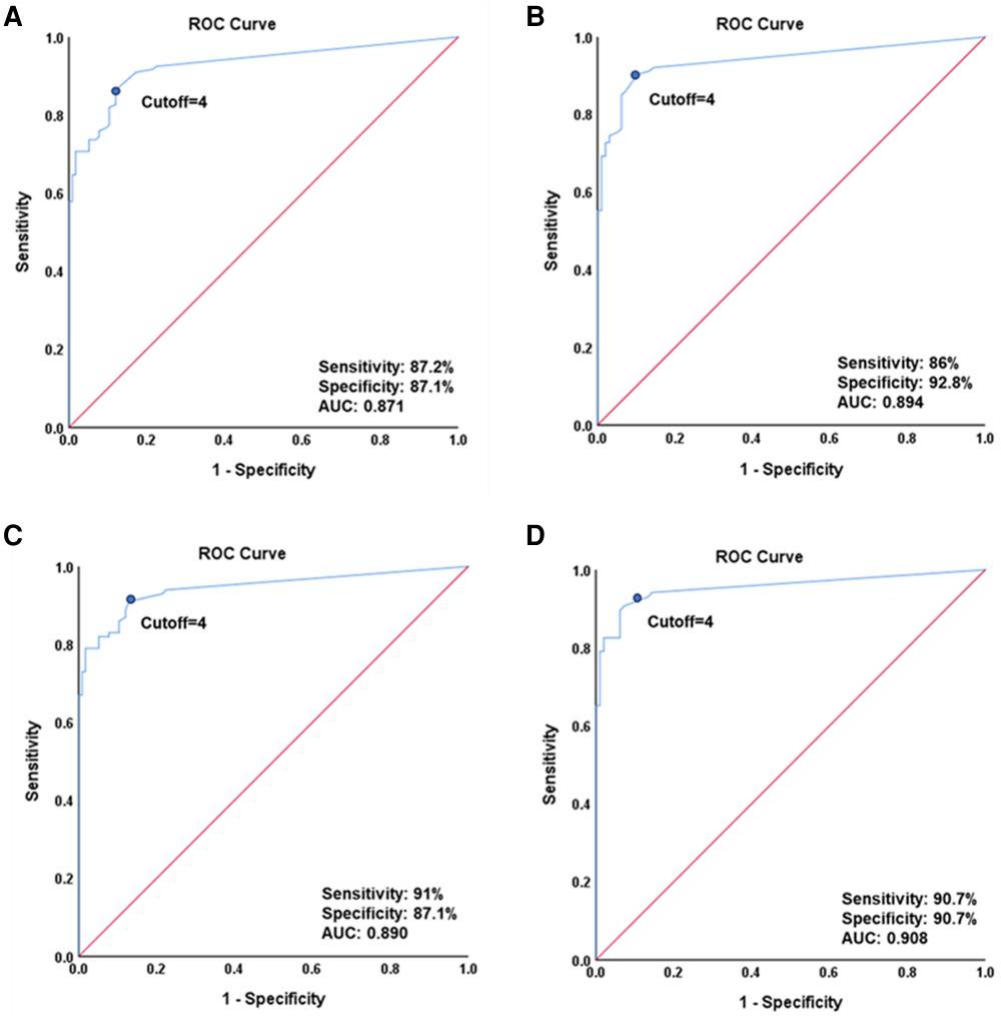
Sensitivity, specificity and AUC of NTS when cutoff is 4 sequences against the composite reference standard in all patients (*A*) and in patients without tuberculosis history (*B*); against the microbiological reference standard in all patients (*C*) and in patients without tuberculosis history (*D*). Abbreviations: AUC, area under the curve; NTS, nanopore-targeted sequencing.

Based on the microbiological reference standard, when patients with a prior PTB history were excluded, the specificity of NTS rose from 0.776 (95% CI, 0.687–0.846) to 0.856 (95% CI, 0.766–0.916), without a loss of sensitivity. When 4 *M. tuberculosis*–specific sequence reads were defined as the optimal cutoff for a positive NTS result, the specificity rose to 0.907 (95% CI, 0.827–0.954) and the sensitivity decreased from 0.942 (95% CI, 0.863–0.978) to 0.907 (95% CI, 0.820–0.956). The corresponding AUC increased to 0.908 (Figure 4).

A subgroup of 40 patients with microbiologically confirmed PTB were excluded from the main analysis due to ongoing antituberculosis treatment at the time of sample collection from 2 to 12 months. Among them, NTS detected *M. tuberculosis* in 34 cases (85%), higher than Xpert (n = 13, 32%) and MGIT 960 (n = 3, 7.5%).

## DISCUSSION

In this study, we systematically evaluated the diagnostic performance of NTS for PTB in patients with smear-negative sputum. Our findings demonstrate that the sensitivity of NTS against either microbiological or composite reference standards was consistently higher than that of Xpert MTB/RIF and MGIT 960 culture, which is consistent with previous studies [19, 20].

To avoid underestimating the sensitivity of the evaluated methods, we excluded 40 microbiologically confirmed patients with PTB who were ongoing with antituberculosis therapy at the time of sample collection. However, among these patients, the detection rate of NTS was 85%, considerably higher than Xpert MTB/RIF (32%) and MGIT 960 culture (7.5%). This suggests that antituberculosis therapy may have less impact on the detection capability of NTS.

However, sensitivity came at the cost of specificity. In our study, the specificity of NTS was 0.776 (95% CI, 0.687–0.846), lower than that of Xpert MTB/RIF (0.974 [95% CI, 0.921–0.993]) and MGIT 960 culture (1.000 [95% CI, 0.960–1.000]). Difference in specificity was observed between patients with and without a history of PTB. Previous studies showed that the specificity of Xpert decreased frequently in individuals with a history of PTB [24–26], especially within the past 2 years [4]. Given its higher sensitivity, NTS may be more prone to false-positive results, resulting in the decreased specificity. After we excluded 38 patients with a history of PTB, the specificity of NTS increased from 0.776 (95% CI, 0.687–0.846) to 0.856 (95% CI, 0.766–0.916), whereas the sensitivity remained unchanged. However, because of the limited number of patients with prior PTB history in our study, we could not conclusively determine how long *M. tuberculosis* DNA will persist after treatment nor its impact on subsequent diagnostic results.

According to previous studies, apart from residual nonviable bacilli and their DNA in old tuberculosis lesions, other factors such as nonspecific amplification, laboratory cross-contamination, limitations in bioinformatics analysis [27–29], and inadequate decontamination of bronchoscope [30] could contribute to false-positive results. However, in our study, environment contamination and insufficient bronchoscope decontamination are unlikely to account for all of the false-positive results, because of the strict implementation of laboratory quality control throughout the study. Our implemented quality control procedures include 1 extraction-negative and 1 amplification-negative control in each batch to monitor potential contamination throughout the entire workflow. In addition, we provide regular training and competency assessments for personnel responsible for bronchoscope disinfection, ensuring adherence to recommended guidelines.

Among the 5 cases of NTM-PD, NTS detected both *M. tuberculosis* and NTM sequences in BALF samples. One possible explanation is NTM/MTB coinfection, which may be detected by highly sensitive methods such as NTS but missed by current diagnostic tools. Technical false positivity is another possibility, as some NTS targets (eg, rpoB, 16S rRNA) are shared by NTM and MTB. However, the inclusion of TB-specific genes like hsp65 and gyrB should help limit this risk. Future studies may further improve specificity by incorporating more TB-specific targets or assigning them greater weight in NTS interpretation.

Currently, there is no standardized protocol for NTS, and no published studies have specifically addressed the optimal read-count cutoff for a positive nanopore sequencing result [22]. The WHO Consolidated Guidelines on Tuberculosis, Module 3: Diagnosis (2021) and the accompanying Operational Handbook emphasize that low-level molecular signals should be interpreted in conjunction with clinical findings and other diagnostic information . *M. tuberculosis*, as a highly prioritized pathogen, was considered a positive result even if the read is as low as 1 in prior studies [9, 31]. Therefore, in our study, 1 *M. tuberculosis*–specific sequence read was tentatively set as the threshold for positive NTS results, although its clinical significance remains to be confirmed.

Youden's index was used to identify the optimal threshold for a positive NTS result. When the threshold was set increase to 4 *M. tuberculosis*–specific sequence reads, the specificity of NTS improved from 0.856 (95% CI, 0.766–0.916) to 0.907 (95% CI, 0.827–0.954), and the sensitivity decreased from 0.942 (95% CI, 0.863–0.978) to 0.907 (95% CI, 0.820–0.956), but still remained higher than Xpert. Meanwhile the AUC improved to 0.908.

NTS inherit the advantages of next-generation sequencing and can detect 10,000 pathogens without prediction [22, 32]. In our study, NTS detected 15 PTB patients with co-infection, including *Pseudomonas aeruginosa, Klebsiella pneumoniae*, *Aspergillus fungus*, *Cryptococcus,* and viruses. This means NTS had obvious advantages over other molecular detection techniques in identifying PTB with concurrent infections. NTS can also accurately distinguish between *M. tuberculosis* and nontuberculous mycobacteria, a capability that Xpert MTB/RIF does not provide [33, 34]. NTS has relatively low requirements for testing environments and operators [35, 36], which is incomparable to second-generation sequencing.

There are several limitations in our study. First, differences in sample volume and processing protocols between NTS and Xpert MTB/RIF may have affected diagnostic performance. Specifically, 10 mL of BALF and a centrifugation step were required for NTS compared with 2 mL and no centrifugation for Xpert. Therefore, our comparison predominantly reflects the overall performance of the diagnostic methods rather than a direct comparison between sequencing and PCR-based techniques. Second, the optimal read-count cutoff for positive NTS results was derived from our internal data and requires external validation in independent cohorts. Third, there remains a risk of false-positive NTS results. A prospective multicenter study with extended follow-up is required to validate our findings and to further elucidate the clinical relevance and long-term outcomes of patients who test positive by NTS but negative by culture. It is important to note that clinicians should not rely solely on positive NTS sequencing results, but should integrate comprehensive clinical, radiological, and microbiological information when making treatment decisions.

## CONCLUSION

In summary, NTS shows significant potential as a rapid and highly sensitive diagnostic tool for pulmonary tuberculosis, especially in patients with low bacillary loads or those already receiving therapy. Optimizing the sequence read threshold and incorporating history of TB can help minimize false positives, thereby enhancing the clinical utility of NTS.
